# Development and implementation of a continuing medical education program on non-alcoholic fatty liver disease for primary care practitioners in Europe

**DOI:** 10.3389/fmed.2023.1034626

**Published:** 2023-03-23

**Authors:** Sophia Papadakis, Marilena Anastasaki, Irini Gergianaki, Ger Koek, Juan Mendive, Foteini Anastasiou, Leen Heyens, Montserrat Garcia-Retortillo, Jean Muris, Christos Lionis

**Affiliations:** ^1^Clinic of Social and Family Medicine, School of Medicine, University of Crete, Crete, Greece; ^2^Division of Gastroenterology and Hepatology, Department of Internal Medicine, Maastricht University Medical Center, Maastricht, Netherlands; ^3^School of Nutrition and Translational Research in Metabolism-NUTRIMM, aastricht University, Maastricht, Netherlands; ^4^La Mina Primary Health Care Centre - IDIAP Jordi Gol, Barcelona, Spain; ^5^European Society for Primary Care Gastroenterology, London, United Kingdom; ^6^Faculty of Medicine and Health Sciences, Hasselt University, Hasselt, Belgium; ^7^Hepatology Unit, Hospital del Mar, Barcelona, Spain; ^8^Department of Family Medicine, CAPHRI Research Institute, Maastricht University, Maastricht, Netherlands

**Keywords:** non-alcoholic fatty liver disease, training, primary care, Europe, Greece

## Abstract

**Background:**

Primary care has a crucial role to play in the prevention, early detection, referral, and risk factor management of non-alcoholic fatty liver disease and non-alcoholic steatohepatitis (NAFLD/NASH). In 2021, a team of European collaborators developed a continuing medical education (CME) program on NAFLD/NASH that consolidates evidence and clinical best practices tailored to the primary care setting. This article reports on the methodology used to design and develop the CME and the results of a feasibility study.

**Methods:**

An expert advisory group representing both European specialists and general practitioners supported the design of the CME to be implemented in three European settings (Greece, Spain, and Netherlands). The CME features four training modules and problem-based learning using clinical case studies. The CME was tested regarding feasibility and acceptability among a sample of primary care providers (PCPs) in Greece (*n* = 28) with measurements occurring before, immediately after, and 1 month following the training. Outcome measures included satisfaction with the CME, changes in PCPs’ knowledge, attitudes, confidence, and self-reported clinical practices related to NAFLD/NASH.

**Results:**

The CME is available as an open-access e-learning course on the European Society for Primary Care Gastroenterology education platform^1^ in English, Greek, Spanish, and Dutch. The feasibility study documented high levels of satisfaction, with 96% of PCPs reporting they were extremely or very satisfied with the overall training. Statistically significant increases in PCPs’ confidence in NAFLD/NASH-related clinical practices were documented between the pre- and post-assessments. At the follow-up, 62% of GPs reported that the CME had changed their clinical practices related to NAFLD/NASH to a great extent.

**Conclusion:**

This CME intervention developed by experts and tailored to PCPs in European settings may serve as an asset for increasing knowledge, confidence, and practice behaviors related to NAFLD/NASH.

## Introduction

Chronic liver disease is a major cause of morbidity and mortality worldwide, with non-alcoholic fatty liver disease (NAFLD) and a subtype of NAFLD, non-alcoholic steatohepatitis (NASH), being significant causes of chronic liver disease (1–6). Estimates indicate that the prevalence of NAFLD exceeds 25% in European adults, with a significant rise in the incidence of NAFLD/NASH predicted globally (6, 7). Rates of NAFLD among patients with obesity and/or type 2 diabetes mellitus (T2DM) have been shown to exceed 70% (2, 8). Importantly, NAFLD is associated with disturbed metabolic function and increased incidence of cardiovascular diseases (CVDs), dyslipidemia, insulin resistance, T2DM, and hypertension, which are components of the metabolic syndrome (1, 2, 9–14).

There has been increasing recognition that general practitioners and primary care providers (PCPs) may have a crucial role to play in the prevention, early detection (case finding), and long-term management of the NAFLD spectrum (1, 2, 15, 16). Despite the existence of clinical guidelines, NAFLD and NASH have received insufficient attention to date by primary care providers in Europe and internationally, with a large proportion of cases going undiagnosed or receiving a late diagnosis (1, 2, 6, 17, 18). Addressing the risk of advanced fibrosis in patients with obesity, T2DM, and dyslipidemia is particularly important however is not yet standard practice in European primary care settings (6, 18). There are limited studies that have examined physician knowledge, attitudes and practice patterns, and barriers related to NAFLD/NASH (18–22). A study of PCPs (*n* = 250) found that the screening rates for NAFLD among patients with obesity and diabetes were less than 46% (18). Poor familiarity with NAFLD/NASH guidelines and low confidence in terms of knowledge and skills for addressing NAFLD/NASH and the complexity of the disease have been identified as barriers to patient care for PCPs (18, 20, 21, 23, 24). Available data indicate that most clinicians have not read clinical practice guidelines or received continuing medical education concerning NAFLD/NASH (18, 19, 22). There has been a call for more education of physicians and other healthcare professionals on NAFLD/NASH guidelines by the international medical community including primary care (1, 2, 20, 21, 23, 24). Such training should be brief, accessible, and address identified knowledge gaps and new and emerging research. The recent European Association for the Study of the Liver (EASL)-Lancet Liver Commission report emphasized that training for PCP should, in particular, focus on health promotion and prevention of liver diseases and earlier-stage diagnosis of this disease, indicating that primary care is particularly well-suited to address these areas of clinical practice (1).

In 2021, a team of European collaborators undertook to develop a continuing medical education (CME) program that consolidated evidence, clinical best practices, and case studies tailored to the primary care setting. This article reports on the rigorous methodology undertaken to inform the content and development of a CME intervention in three European settings. We also report on the results of a feasibility study of the CME among a sample of PCPs in Greece to examine the effects of training on providers’ knowledge, attitudes, confidence, and clinical practices related to NAFLD/NASH as well as satisfaction with various aspects of the CME intervention.

## Materials and methods

### Context

This study was undertaken as part of a larger multi-phase European collaborative project entitled *“Implementing and evaluating a clinical care pathway for NALFD/NASH models in Primary Care”* (1).^2^ The project involves five interrelated work packages studying both patient and PCP knowledge and perspectives, developing and testing an educational model of care intervention tailored to primary care practice settings in three European countries (Greece, Netherlands, and Spain).

#### Theoretical framework

The Theory of Planned Behavior was selected as the theoretical framework to guide training intervention design (25, 26). Specifically, the e-learning intervention was designed to increase three intermediary targets known to be associated with intentions and clinical practice behaviors for NAFLD/NASH screening, diagnosis, referral, and co-management: (1) PCPs’ confidence in implementing the behaviors (perceived behavioral control); (2) PCPs’ attitudes regarding the importance of clinical behaviors related to NAFLD/NASH; and (3) perceived clinical norms related to the role of primary care in NAFLD/NASH. PCPs’ confidence in various clinical practices was prioritized and techniques used to increase PCP confidence were included in the CME. These included the following: (1) increasing knowledge of evidence-based NAFLD/NASH guidelines; (2) increasing skills through discussing clinical scenarios representative of primary care; and (3) modeling practice behaviors.

### Part 1: Development of CME module on NAFLD/NASH for primary care practice

#### Procedures

##### Step 1: Scan and synthesize training assets and relevant literature

The project team conducted an initial review and a synthesis of current literature and training assets and formulated evidence-based statements and suggestions for the themes to be addressed. A summary report with preliminary conclusions and new emerging questions was drafted and circulated to project collaborators.

##### Step 2: Expert advisory group

A European expert advisory group supported the design of the CME. The expert panel represented specialists, general practitioners, academicians, and health officers with documented interest and experience in the field of NAFLD/NASH as well as PC. Expert panel members provided scientific and clinical expertise to the design and development of the CME program. Two online meetings of the expert panel were held in November and December 2020. Expert panel members included representation from European Associations, including the European Society for Primary Care Gastroenterology (ESPCG) and EASL. *The expert panel discussed the* evidence base and existing knowledge on barriers and facilitators to PCPs’ implementation of NAFLD/NASH clinical practice guidelines and the guiding theoretical framework and supported the development of the learning objectives, content outline, and key messages for the CME intervention. The consensus during the meeting was initiated by discussion questions, a round table discussion with expert panel members, followed by a plenary session and consensus-seeking.

##### Step 3: CME content development and review

A working group led by the Clinic of Social and Family Medicine at the University of Crete (UoC), Maastricht University, La Mina Primary Health Care Centre and the ESPCG led content development. The outcomes and direction identified as part of the expert panel meeting were triangulated with information gained from other sources and literature. Training content, including PowerPoint slides and speaker notes for a four-module CME program, was drafted by the working group. Problem-based learning using clinical case studies was a major feature of the training program’s design. Furthermore, guidance from the recently published NAFLD patient guideline was incorporated into the training (27). Each partner organization led the development of a case study focusing on PCPs scenarios to demonstrate the clinical application of knowledge and skills addressed in the training. Several rounds of the review were undertaken by members of the working group to refine the course materials. The training package was initially developed in English and then translated to Greek, Spanish, and Dutch by local teams for use in local feasibility testing. Country-level clinical leaders recorded the CME as e-learning in the local language using Zoom. The ESPCG central office was responsible for coordinating these tasks and allocating the different translated training packages on its e-learning platform.

### Part 2: Feasibility testing of the NAFLD/NASH training intervention

#### Design

A before-and-after, cross-sectional feasibility study was conducted. An online survey was conducted among participating PCP immediately before, immediately following, and 1 month after exposure to the e-learning training.

#### Setting and population

The sample size selected represented a convenience sample of PCPs from established lists of GPs in Crete. A random sample of GPs (*n* = 50) from the island of Crete in Greece were selected from existing listings of GPs in the pre-defined geographic areas. The sample represents approximately 50% of GPs in the geographic region and as such felt to be representative for this pilot evaluation. The sample was stratified by years of experience (< 10 or ≥ 10 years), geographic setting (urban/suburban/rural area), and work in the private or public domain to allow for group representation. A UoC researcher who is not involved in the present study prepared a computer-generated random number sequence to select GPs within the stratification groups. To be eligible to participate in the study, individuals should be (1) licensed GPs, (2) currently working in a general practice setting, (3) willing to complete the e-learning training intervention in the next 30-days, and (4) have access to a computer with internet to complete the online training.

#### Procedures

The randomly selected GPs were contacted by email (or postal mail) and invited to take part in the study and a telephone follow-up will be placed at each GP’s office. Participating GPs received an email with information on accessing the training intervention. GPs completed the online pre-training survey before accessing the CME intervention. Participants were exposed to a four-module, 90-min CME intervention. The option was provided to participate in a live webinar and/or open-access e-learning. The post-training survey was implemented immediately following the completion of the e-learning module. An email was sent to GPs 1 month following the completion of the e-learning with a link to the follow-up survey. The 1-month time frame for follow-up was selected as being suitable for assessing post-training changes to clinical practice and attitudes. Up to three reminders were placed at 1, 2, and 4 weeks to GPs to remind them to complete the surveys before classifying them as non-respondents. As such, data represent a time frame for measurement of 4 to 8 weeks post-training.

#### Ethics, informed consent, and confidentiality

The study was approved by the University of Crete Research Ethics Committee, as well as the local ethical and research committees of each country, and conducted according to the ethical guidelines of the Helsinki Declaration. All participating primary care providers provided written informed consent.

#### Outcome measures

Outcome measurement included provider knowledge, attitudes, confidence, and clinical behaviors, as well as measures of satisfaction and value of the training program (Supplementary Table 1). A review of the literature was conducted to identify published survey instruments that assessed the tested outcomes (18, 22). These survey instruments were reviewed by our investigative team and modified and supplemented to align with the learning targets of the e-learning intervention program (2).

##### Primary care providers’ satisfaction with training and commercial bias

Primary care providers were asked, *“Overall how satisfied were you with the online NASH CME you completed?,”* with response options on a 1–5 Likert scale (1, extremely satisfied; 2, very satisfied; 3, satisfied; 4, somewhat satisfied; and 5, not at all satisfied). We also asked participants using the same Likert scale how satisfied they were with the following aspects of the training: the content of the training program, the quality of the presentation, the case studies used, and the duration of the training. PCPs were asked using free text responses about what they liked best about the training and what they liked least, as well as, suggestions for improving the training intervention. Finally, participants were asked to assess commercial bias (*“Did you feel the training was free of commercial bias?”* Response options: 1 Yes and 2 No).

##### Primary care providers’ confidence (perceived behavioral control)

Eight items were used to assess PCP’s confidence in NAFLD/NASH knowledge and clinical practices related to screening, detection, referral, and treatment. Confidence was evaluated on a 1–5 scale using the question: *“On a scale of 1 to 5, how would you describe your self-confidence in the following areas?: (1 not feeling confident at all and 5 feeling extremely confident).”*

##### Primary care providers’ knowledge

Knowledge related to NAFLD/NASH was assessed using seven multiple-choice questions which assessed participant’s knowledge of the content covered in each of the four training modules and addressed the prevalence of NAFLD; NAFLD/NASH risk factors; NAFLD-related extrahepatic diseases and conditions; and screening, assessment, referral, and co-management.

##### NAFLD/NASH-related attitudes and clinical norms

Two Likert-scale questions (scale 1–5) assessed PCPs’ attitudes about the importance of NAFLD/NASH in primary care and their clinical practice. The additional three items evaluated perceived clinical norms related to the role of PCPs and specialists in the management of NAFLD/NASH.

##### Non-alcoholic fatty liver disease/NASH-related clinical practices

Seven items were used to assess current clinical practice related to NAFLD/NASH at baseline and the 1-month follow-up. Specifically, practitioners will be asked if they had seen and diagnosed NAFLD/NASH in the past year, if they screened patients with obesity and diabetes for NAFLD, if they used non-invasive testing in patients at high-risk groups, and if they referred patients with NAFLD to liver specialists. In addition, at the 1-month follow-up, PCPs were asked *“Would you say that the training program influenced your own clinical practices in relation to NAFLD/NASH? (response options: to a great extent, somewhat, a little, not at all.)”*

### Statistical analysis

Descriptive statistics were calculated, and univariate/multivariate models were used to examine changes in outcome measures after exposure to the e-learning program McNemar’s tests or marginal homogeneity tests (categorical variables), and Wilcoxon’s signed rank tests (continuous variables) were used for pairwise comparisons with a significance level of 0.05. All analyses were conducted using IBM SPSS Statistics for Windows, Version 28.0.

## Results

### Identification of PCP learning needs

**Figure 1:**
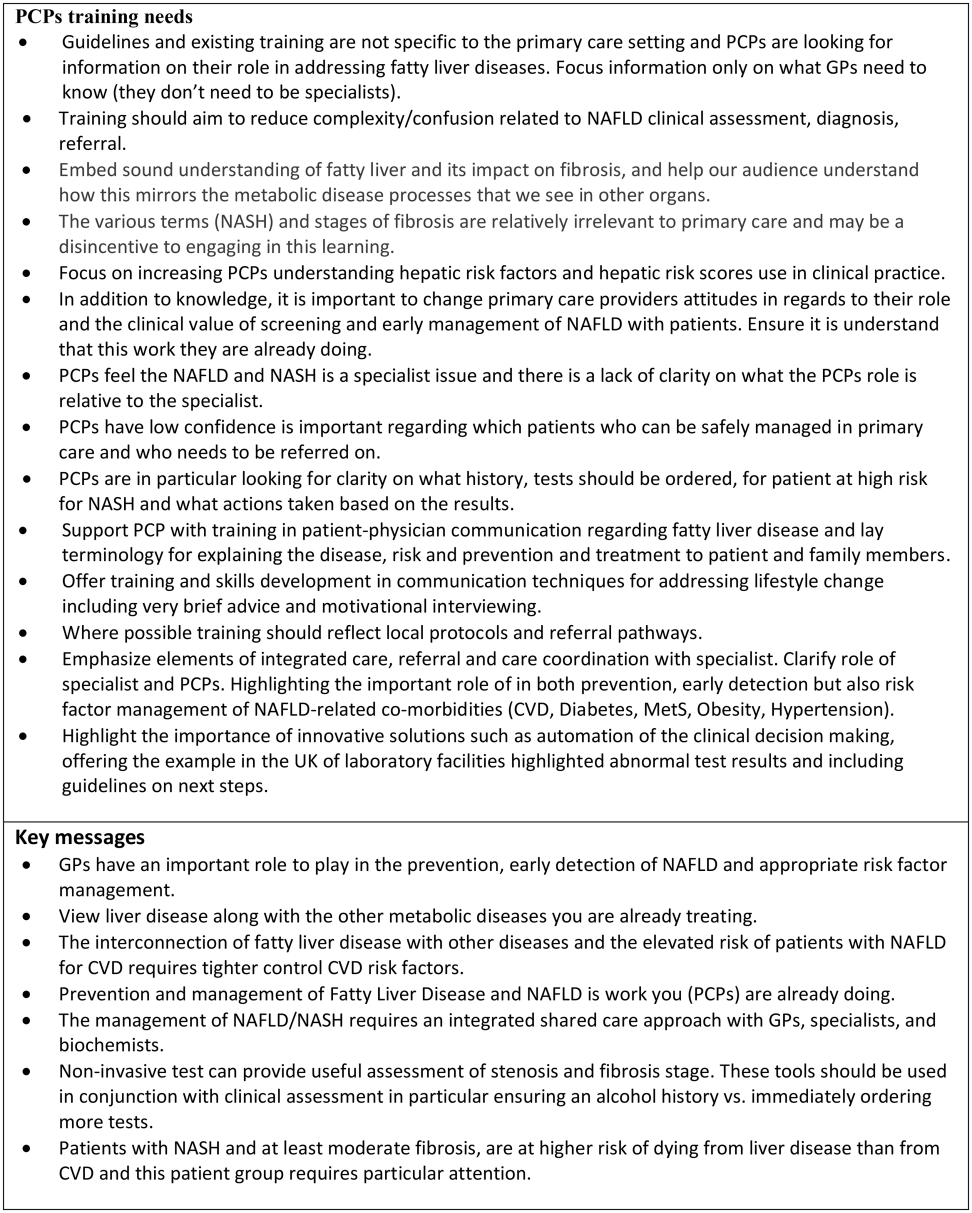
Summary of expert panel assessment of primary care providers (PCPs) training needs and key messages related to Fatty Liver, non-alcoholic fatty liver disease (NAFLD)/non-alcoholic steatohepatitis (NASH).

Figure 1 summarizes the expert panel’s assessment of PCPs’ training needs as well as key messages to be used to guide the training interventions design. Importantly, these key messages represent the opinion of the expert panel comprising leading experts.

### Description of training intervention

**Figure 2:**
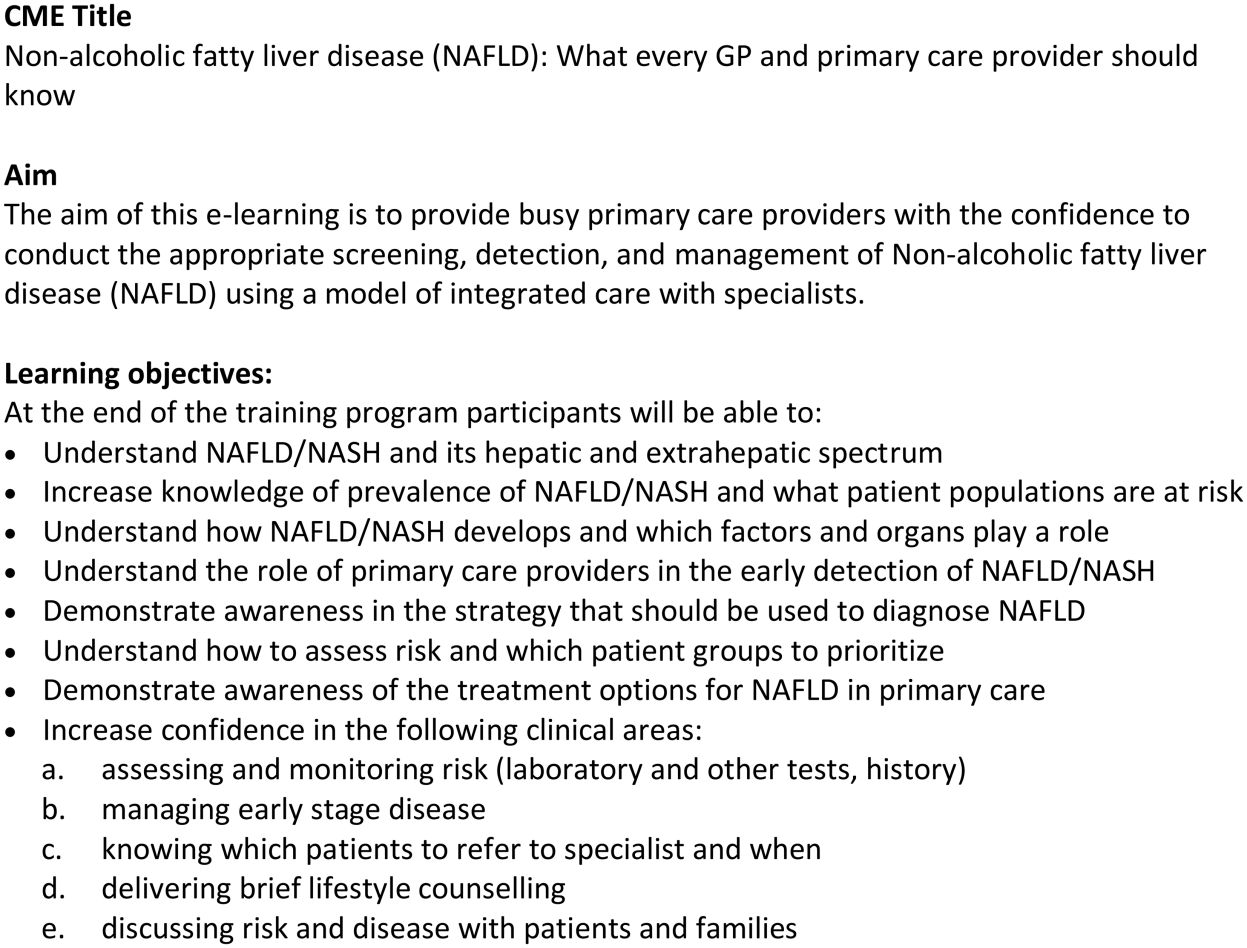
Title, aim, learning objectives, and content outline of the CME intervention.

**Figure 3:**
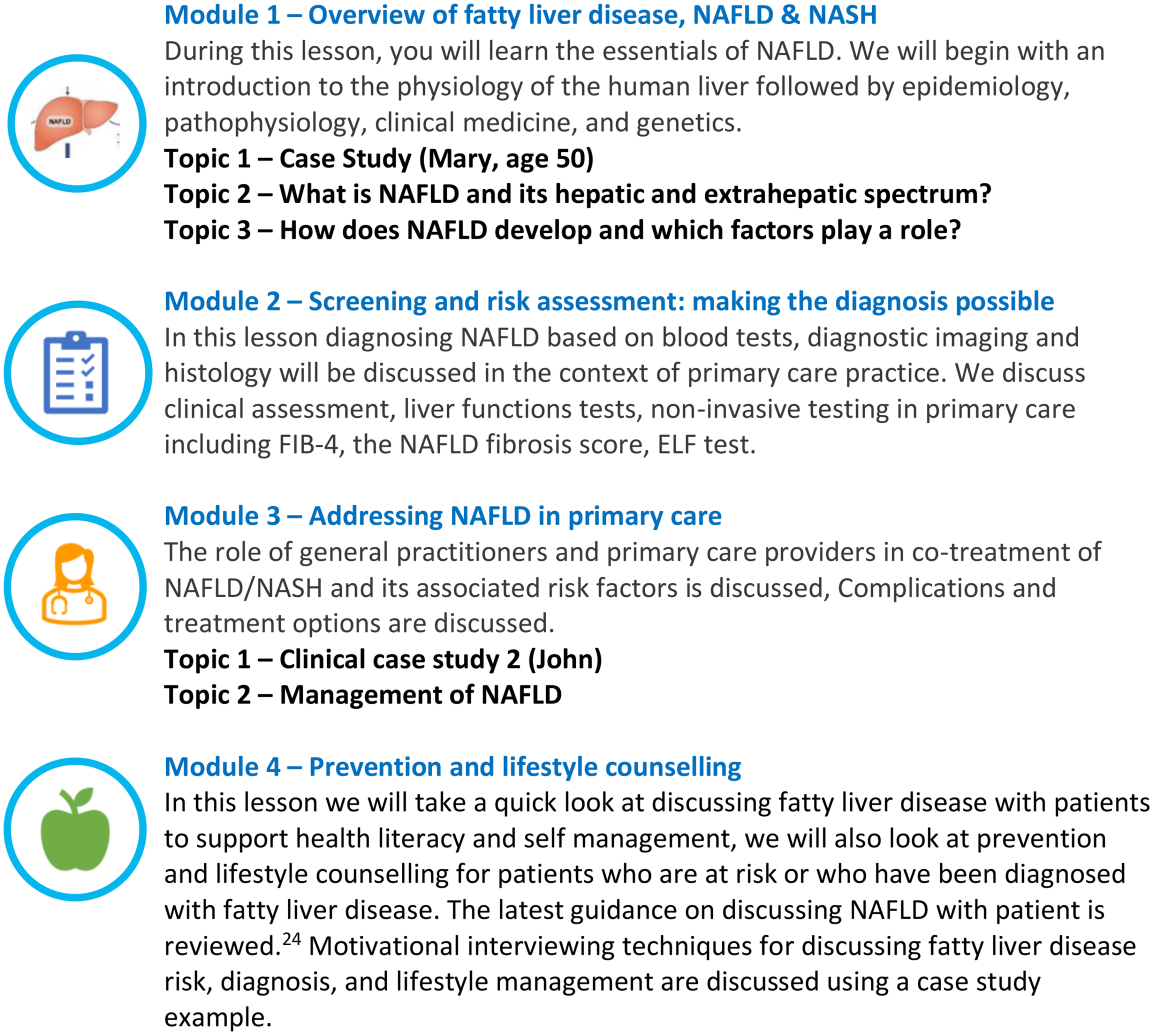
Continuing medical education (CME) content outline.

The aims and learning objectives are described in Figure 2, and the content outline for the four-module training is presented in Figure 3. The CME is hosted as an open-access e-learning course on the ESPCG education platform^3^ in four languages (English, Greek, Spanish, and Dutch).

### Feasibility study

A total of 28 general practitioners participated in the CME intervention feasibility study, representing 58% (*n* = 28/50) of invited PCPs. The primary reasons for non-participation were a lack of time and competing demands with clinical practice responsibilities. The study occurred during the COVID-19 pandemic and lockdown period and PCPs reported that this directly influenced participation rates. Pre-training data were available for 100% of PCPs, and post-training data were available for 96.5% (*n* = 27/28) and 50% (*n* = 14/28) of participating PCPs at the post-training and 1-month follow-up assessment, respectively.

#### Sample characteristics

The characteristics of participating PCPs (42.9% male; mean 18.3 SD 5.9 years in clinical practice) are presented in Table 1. Most participants were between 30 and 50 years of age with representation from urban and rural practice settings. Among respondents, 75% reported that they had not completed a previous CME on NAFLD/NASH.

**Table 1:** Characteristics of general practitioners who participated in the feasibility study (*n* = 28).

Characteristic	No. (%)
*Age*	
<30	2 (7.1)
30–39	13 (46.4)
40–49	12 (42.9)
50–59	1 (3.6)
60–69	0 (0)
70+	0 (0)
*Gender*	
Male	12 (42.9)
Female	16 (57.1)
*Geography*	
Urban	12 (42.9)
Sub-urban	0 (0)
Rural	16 (57.1)
Years in medical practice, mean (SD)	18.3 (5.9)
min-max	5–28
*Previous CME on NAFLD/NASH*	
Yes	7 (25.0)
No	21 (75.0)
*I have read the clinical practice guidelines on the management of NAFLD*	
Yes	7 (25.0)
No	21 (75.0)

#### Satisfaction

High levels of satisfaction were documented with 96.3% of GPs reporting that they were extremely or very satisfied with the training overall. The majority of participants were extremely satisfied or very satisfied with the content, quality of presentation, case studies, and duration of the training (Table 2). The duration of the training was either satisfactory or somewhat satisfactory for approximately 15% of the participants. This was elaborated on in the free text comments which indicated that some participants found the training was too short in duration and should be expanded. Related remarks in regard to the pace of the training were also received. The majority (95%) of respondents identified that they felt the training was free of commercial bias. There was consistent feedback from participants’ free text comments that the case studies were valued and that they felt the training was valuable.

**Table 2:** Satisfaction with continuing medical education (CME) and assessment of commercial bias (*n* = 27).

Measure	No.	%
*Overall*		
Extremely satisfied	12	44.4
Very satisfied	14	51.9
Satisfied	1	3.7
Somewhat unsatisfied	0	0
*Content of the training program*		
Extremely satisfied	14	51.9
Very satisfied	12	44.4
Satisfied	1	3.7
Somewhat unsatisfied	0	0
*Quality of the presentation*		
Extremely satisfied	14	51.9
Very satisfied	12	44.4
Satisfied	1	3.7
Somewhat unsatisfied	0	0
*Case studies used*		
Extremely satisfied	11	40.7
Very satisfied	16	59.3
Satisfied	0	0
Somewhat unsatisfied	0	0
*Duration of the training*		
Extremely satisfied	8	29.6
Very satisfied	15	55.6
Satisfied	3	11.1
Somewhat unsatisfied	1	3.7
*Free of commercial bias*		
Yes	26	95.0
No	1	5.0

#### Primary care providers’ confidence

Before the training, low-to-moderate levels of confidence in NAFLD/NASH knowledge and skills were documented among the PCPs sampled with the average score being 3 out of 5 for all eight domains assessed. The lowest levels of PCP confidence were reported for NAFLD screening and the use of non-invasive tests. Immediately, following exposure to the CME, significant increases in PCPs’ confidence in NAFLD/NASH-related clinical practices were documented in all eight domains (Table 3). Observed differences remained significant at the 1-month follow-up.

**Table 3:** Changes in NAFLD/NASH-related confidence pre- and post-training and 1-month following exposure to the CME intervention among primary care providers who participated in the feasibility study.

Measure	Pre-training (T1) (*n* = 28)	Post-training (T2) (*n* = 27)	1-month FU (T3)(*n* = 14)	Value of p^*^ T1 *vs* T2	Value of p^*^ T1 *vs* T3
Knowledge: NAFLD/NASH	2.7	3.5	4.0	<0.05	<0.001
Knowledge: NAFLD/NASH risk factors	3.0	3.9	4.1	<0.001	<0.001
Screening for NAFLD/NASH	2.5	3.7	3.9	<0.001	<0.001
Use of non-invasive screening tools	2.4	3.8	3.9	<0.001	<0.001
Which patient to refer to a specialist	2.6	3.8	3.9	<0.001	<0.001
When to refer patients to a specialist	2.7	3.7	3.9	<0.001	<0.001
Treatment of NAFLD	2.7	3.6	4.1	<0.001	<0.001
Co-deliver treatment with specialists	2.6	3.8	3.6	<0.001	<0.001

Assessment question: On a scale of 1–5, how would you describe your self-confidence in the following areas [Response options:(1 = not very confident, 5 = extremely confident]. *value of *p* calculated based on a sample of providers for which data were available at both time points being compared.

#### Primary care providers’ knowledge, attitudes, and clinical norms

Baseline knowledge related to NAFLD/NASH among participating PCPs was moderate. A mixed effect was documented in NAFLD-NASH knowledge following exposure to the CME (Supplementary Table 2). Positive changes were documented in three of the seven knowledge items evaluated. While positive changes were documented in some of the PCPs’ attitudes and clinical norms domains assessed between the pre- and post-assessments, these were not statistically significant (Supplementary Table 3**)**.

#### Non-alcoholic fatty liver disease/NASH clinical practices behaviors

At the follow-up, 62.4% of GPs reported that the CME had changed their clinical practices related to NAFLD/NASH to a great extent. A statistically significant difference was observed between pre and follow-up assessments in terms of the reported proportions of patients screened (Table 4).

**Table 4:** Primary care providers in NAFLD/NASH-related clinical practice behaviors norms pre- and 1-month following feasibility testing of CME intervention.

Clinical practice behaviors	Pre-training % (*n*)	1-month FU % (*n*)	Value of p T1 *vs* T3
*Would you say the NASH e-learning training has influenced your own clinical practices related to NASH?*			
To a great extent	–	64.3 (9)	–
Somewhat		28.6 (4)	
A little		7.1 (1)	
Not at all		0 (0)	
*Do you screen patients with obesity or diabetes mellitus for NAFLD?*			
Yes	64.3 (18)	78.6 (11)	0.250
No	35.7 (10)	21.4 (3)	
*What percentage of your patients with obesity and/or diabetes mellitus did you screen for NAFLD during the past year?*			
All	14.3 (4)	7.1 (1)	0.035
>50%	17.9 (5)	35.7 (5)	
<50% but a lot	46.4 (13)	50.0 (7)	
None	21.4 (6)	7.1 (1)	
*Do you refer patients with NAFLD to a specialist (gastroenterologist)?*			
Yes	64.3 (18)	64.2 (9)	0.655
No	35.7 (10)	35.7 (5)	

Pre-training data available for n = 28 primary care providers.

1-month follow-up data available for n = 14 primary care providers.

*p*-Values calculated based on data available for PCPs with data at both time points.

## Discussion

### Main findings and general discussion

From our project, we have understood that there is an urgent need to design, disseminate, and evaluate CME interventions on NAFLD/NASH that are tailored to address the role of PCPs in European countries. Training interventions should be available in the local language and reflect the local clinical context to maximize uptake. To address this challenge, a European team representing hepatology specialists, general practitioners, nurses, and academic professionals sought to consolidate available evidence into a CME to be disseminated *via* the ESPCG’s e-learning platform, as well as, local country-level training networks. The CME sought to increase PCPs’ confidence in screening and managing patients at high risk for NASH, influence PCPs’ attitudes and skills related to primary care’s role in the early detection and screening of NAFLD/NASH, and promote a new conversation style to address patients’ risk perceptions. The feasibility study documented high rates of satisfaction with the CME programme. Significant increases in PCPs’ confidence in NAFLD/NASH-related clinical practices were documented between the pre- and post-assessments. Notably, following exposure to the training, GPs reported that the CME had changed their clinical practices related to NAFLD/NASH to a great extent. The evaluation documented that eLearning had a mixed effect on PCP’s NAFLD/NASH-related knowledge suggesting that additional attention is needed in ensuring learning objectives for key knowledge areas are achieved. Specifically, knowledge related to the use of non-invasive testing in primary care including FIB-4, the NAFLD fibrosis score, ELF tests, and recommendations for lifestyle change was modest at both the pre- and post-evaluations.

There are several important features of the CME intervention that should be highlighted. This CME was explicitly designed for PCP, and the scope of the training content was selected to cover the general practitioner and other primary care team members’ role in prevention, early detection, and screening, as well as, address referral, communication, and co-management with specialists. While there have been CME interventions disseminated on NAFLD, they have not necessarily been specific to the role of PCPs in NAFLD and NASH. The training intervention was grounded in the latest international guidance, specifically focussed on non-invasive testing that would be accessible in European primary care practice settings. Screening pathways were presented to increase clinicians’ confidence in using bio-markers and non-invasive testing to support clinical decision-making. The training sought to address vital questions our advisory group felt were limiting updates of clinical practice guidelines. The CME features three case studies that reflect clinical scenarios commonly seen in primary care settings. These case studies were designed to show the application of clinical practice guidelines to real-world scenarios and support problem-based learning. Finally, the CME sought to promote the development of new skills for communicating with patients on risks associated with fatty liver disease based on recent guidance published on patient literacy for NAFLD (27). The training also addresses motivational interviewing techniques for addressing NAFLD in the context of a busy primary care practice and discussing risk factor management and lifestyle factors with patients.

To our knowledge, there has been one previously published report evaluating the effects of a training program designed to increase GPs’ knowledge of NAFLD (24). This small study involving 56 GPs in Italy assessed knowledge and practice behaviors before, immediately after, and 4 months after an educational training workshop was completed. The training resulted in improvement in some but not all knowledge areas assessed and also improvement in practice behaviors.

### Strengths and limitations

A rigorous methodology was undertaken to inform and develop the CME intervention and comprehensive evaluation was used to evaluate the effects of the interventions on outcomes of interest. The evaluation tools developed may serve as an asset for future evaluations of provider confidence and behaviors related to NAFLD/NASH and the evaluation of similar training-based interventions. Our feasibility study also had several limitations. The feasibility study was limited to a small number of GPs in one European setting. There was a significant loss to follow-up and results are based on self-reported data, which is subject to bias. Evaluations of the CME using the same methodologies are underway in Spain and Netherlands and will expand the evaluation of the CME to include three European settings and will serve to generate data to benchmark baseline knowledge and confidence and compare the response to training intervention in these three settings. Future studies may wish to consider the validation of PCP self-reported data with chart abstraction or another form of data.

### Implications to practice and research

The recently published EASL-Lancet Liver Commission report states “*primary care and community health-care settings have a crucial part to play in outreach, referring and filtering patients with benign or irrelevant abnormalities in liver blood tests from patients at risk of progressive fibrosis, aided by technology in promoting streamlined care, automated investigation in response to mild abnormalities, and increased access to second line—and second generation—fibrosis testing”* (1). For PCP to fulfill this role, high-quality training resources, such as that reported here, are needed and support disseminating these resources within European PCP settings will be equally as important (17). The new training assets reported on in this report may serve this purpose. There is preliminary evidence from the present feasibility study that the training was well-received and influenced outcomes of interest, including clinical practice behaviors. In addition to the ESPCG e-learning reported in this article, the European Association for the Study of Diabetes (EASD) has also recently published e-learning on NAFLD.^4^ The increased interest in national organizations in offering such training indicates the need for such training and support.

While the availability of such training is crucial, to be successful in influencing practice behaviors, it will require promotion among the European primary care practice community including the involvement of country-level and local training networks. Evaluation of the effects of training interventions such as that reported in our feasibility testing will be invaluable in ensuring that training is meeting learning objectives and informing the design of future updates to training and informing future clinical practice guidelines. While training is a necessary part of increasing rates of screening and treatment in primary care settings, training interventions alone are likely to be insufficient and should be complemented by interventions that automate screening, straightforward algorithms for screening and referral, new models of collaborative care, and changes to clinical practice norms including incentives (1). This project was part of a larger European collaborative project looking at enhancing models of care related to NAFLD/NASH in primary care, the results of which are forthcoming. In Greece, retraining the primary care workforce is one of the priorities of the current primary care reform agenda, and in a post-COVID-19 period, e-learning will be a key vehicle for supporting CME. Training assets such as the present e-learning have the opportunity to be refined and disseminated nationally as part of this imitative. At present, early identification and risk factor management including lifestyle change (i.e., dietary and weight loss) are the available treatment options to clinicians and the focus is on the CME tested. It is important to recognize that lifestyle interventions have been a challenging area of patient care with varying levels of success in terms of patient ability to make desired changes and meet clinically relevant lifestyle targets. Advancements in treatment options including medications are needed.

Interestingly, while the advisory committee had identified the need to be as brief as possible with training to enhance uptake by busy PCP, qualitative and quantitative data from the feasibility study indicated that a proportion of PCPs felt the training should be longer than the 90 min offered *via* this CME. This finding should be validated but may be indicative of a knowledge and confidence gap among PCPs related to NAFLD. Likewise, while significant increases in confidence and methods were observed as part of the feasibility study, there is potential for even greater increases in both confidence and knowledge; methods for achieving this *via* CME or booster training, or other means, should be examined.

### Conclusion

This CME intervention and the evaluation instruments, developed by experts and tailored to PCPs in Europe, may serve as an asset for increasing knowledge, confidence, and practice behaviors related to NAFLD/NASH. Expanding the availability of training interventions to other languages and supporting the dissemination of available training assets to PCPs in Europe should be a focus of future work.

## Data availability statement

The original contributions presented in the study are included in the article/Supplementary material, further inquiries can be directed to the corresponding author.

## Ethics statement

The study was approved by involving human participants were reviewed and approved by University of Crete Research Ethics Committee. The participants provided their written informed consent to participate in this study.

## Author contributions

SP, MA, and CL contributed to the study conception and design and manuscript writing. SP and MA performed the statistical analysis. GK, SP, CL, IG, JMe, LH, MG-R, JMu, and FA contributed to the design and development of the CME program. IG coordinated data collection activities. JMe, GK, IG, JMu, LH, and MG-R conducted a critical revision of the article for important intellectual content. All authors read and approved the final version of the manuscript.

## Funding

This study was funded by an unrestricted research grant from Gilead Science Inc. (NASH Models of Care grant number IN-EU-989-5753). The study funders had no role in any aspect of study design, conduction, data analysis, interpretation of results, and reporting.

## Conflict of interest

The authors declare that the research was conducted in the absence of any commercial or financial relationships that could be construed as a potential conflict of interest.

## Publisher’s note

All claims expressed in this article are solely those of the authors and do not necessarily represent those of their affiliated organizations, or those of the publisher, the editors and the reviewers. Any product that may be evaluated in this article, or claim that may be made by its manufacturer, is not guaranteed or endorsed by the publisher.

## Footnotes

^2^ www.nash.med.uoc.gr

^3^ https://www.espcg.eu/courses/nafld-elearning-module-for-primary-care/

^4^ https://easd-elearning.org/courses/nafld/

## References

[1] Karlsen TH Sheron N Zelber-Sagi S Carrieri P Dusheiko G Bugianesi E . The EASL-lancet liver commission: protecting the next generation of Europeans against liver disease complications and premature mortality. Lancet. (2022) 399:61–116. doi: 10.1016/S0140-6736(21)01701-3, PMID: 34863359

[2] European Association for the Study of the Liver (EASL) . European Association for the Study of diabetes (EASD); European Association for the Study of obesity (EASO). EASL-EASD-EASO clinical practice guidelines for the management of non-alcoholic fatty liver disease. J Hepatol. (2016) 9:65–90. doi: 10.1159/000443344, PMID: 27055256 PMC5644799

[3] Ofosu A Ramai D Reddy M. Non-alcoholic fatty liver disease: controlling an emerging epidemic, challenges, and future directions. Ann Gastroenterol. (2018) 31:288–95. doi: 10.20524/aog.2018.0240, PMID: 29720854 PMC5924851

[4] Mokdad AA Lopez AD Shahraz S Lozano R Mokdad AH Stanaway J . Liver cirrhosis mortality in 187 countries between 1980 and 2010: a systematic analysis. BMC Med. (2014) 12:145. doi: 10.1186/s12916-014-0145-y, PMID: 25242656 PMC4169640

[5] Marcellin P Kutala BK. Liver diseases: a major, neglected global public health problem requiring urgent actions and large-scale screening. Liver Int. (2018) 38:2–6. doi: 10.1111/liv.13682, PMID: 29427496

[6] Sarwar R Pierce N Koppe S. Obesity and nonalcoholic fatty liver disease: current perspectives. Diabetes Metab Syndr Obes. (2018) 11:533–42. doi: 10.2147/DMSO.S146339, PMID: 30288073 PMC6163009

[7] Cholongitas E Pavlopoulou I Papatheodoridi M Markakis GE Bouras E Haidich AB . Epidemiology of nonalcoholic fatty liver disease in Europe: a systematic review and meta-analysis. Ann Gastroenterol. (2021) 34:404–14. doi: 10.20524/aog.2021.0604, PMID: 33948067 PMC8079871

[8] de Alwis NM Day CP. Non-alcoholic fatty liver disease: the mist gradually clears. J Hepatol. (2008) 48:S104–12. doi: 10.1016/j.jhep.2008.01.009, PMID: 18304679

[9] Armstrong MJ Adams LA Canbay A Syn WK. Extrahepatic complications of nonalcoholic fatty liver disease. Hepatology. (2014) 59:1174–97. doi: 10.1002/hep.26717 24002776

[10] Chalasani N Younossi Z Lavine JE Charlton M Cusi K Rinella M . The diagnosis and management of nonalcoholic fatty liver disease:practice guidance from the American Association for the Study of Liver Diseases. Hepatology. (2018) 67:328–57. doi: 10.1002/hep.29367 28714183

[11] Muzurović E Mikhailidis DP Mantzoros C. Non-alcoholic fatty liver disease, insulin resistance, metabolic syndrome and their association with vascular risk. Metabolism. (2021) 119:154770. doi: 10.1016/j.metabol.2021.154770, PMID: 33864798

[12] Bedogni G Gastaldelli A Foschi FG. Fatty liver, cardiometabolic disease and mortality. Curr Opin Lipidol. (2020) 31:27–31. doi: 10.1097/MOL.0000000000000652 31789677

[13] Caussy C Aubin A Loomba R. The relationship between type 2 diabetes, NAFLD, and cardiovascular risk. Curr Diab Rep. (2021) 21:15. doi: 10.1007/s11892-021-01383-7, PMID: 33742318 PMC8805985

[14] Vernon G Baranova A Younossi ZM. Systematic review: the epidemiology and natural history of non-alcoholic fatty liver disease and non-alcoholic steatohepatitis in adults. Aliment Pharmacol Ther. (2011) 34:274–85. doi: 10.1016/S0973-6883(12)60102-9, PMID: 21623852

[15] Williams R Alexander G Armstrong I Baker A Bhala N Camps-Walsh G . Disease burden and costs from excess alcohol consumption, obesity, and viral hepatitis: fourth report of the lancet Standing commission on liver disease in the UK. Lancet. (2018) 391:1097–107. doi: 10.1016/S0140-6736(17)32866-0, PMID: 29198562

[16] Tsochatzis EA Newsome PN. Non-alcoholic fatty liver disease and the interface between primary and secondary care. Lancet Gastroenterol Hepatol. (2018) 3:509–17. doi: 10.1016/S2468-1253(18)30077-3. PMID: 29893235

[17] Standing HC Jarvis H Orr J Exley C Hudson M Kaner E. Barbara Hanratty. Br J Gen Pract. (2018) 68:e743–9. doi: 10.3399/bjgp18X699377, PMID: 30249611 PMC6193778

[18] Said A Gagovic V Malecki K Givens ML Nieto FJ. Primary care practitioners survey of non-alcoholic fatty liver disease. Ann Hepatol. (2013) 12:758–65. doi: 10.1016/S1665-2681(19)31317-1, PMID: 24018493

[19] Kallman JB Arsalla A Park V Dhungel S Bhatia P Haddad D . Screening for hepatitis B, C and nonalcoholic fatty liver disease: a survey of community-based physicians. Aliment Pharmacol Ther. (2009) 29:1019–24. doi: 10.1111/j.1365-2036.2009.03961.x, PMID: 19220207

[20] Polanco-Briceno S Glass D Stuntz M Caze A. Awareness of nonalcoholic steatohepatitis and associated practice patterns of primary care physicians and specialists. BMC Res Notes. (2016) 9:157. doi: 10.1186/s13104-016-1946-1, PMID: 26969270 PMC4788895

[21] Sebastiani G Ramji A Swain MG Patel K. A Canadian survey on knowledge of non-alcoholic fatty liver disease among physicians. Can Liver J. (2021) 4:82–92. doi: 10.3138/canlivj-2020-0033, PMID: 35991764 PMC9204942

[22] Matthias AT Fernandopulle ANR Seneviratne SL. Survey on knowledge of non-alcoholic fatty liver disease (NAFLD) among doctors in Sri Lanka: a multicenter study. BMC Res Notes. (2018) 11:556. doi: 10.1186/s13104-018-3673-2, PMID: 30075812 PMC6076419

[23] Standing HC Jarvis H Orr J Exley C Hudson M Kaner E . GPs’ experiences and perceptions of early detection of liver disease: a qualitative study in primary care. Br J Gen Pract. (2018) 68:e743–9. doi: 10.3399/bjgp18X699377, PMID: 30249611 PMC6193778

[24] Grattagliano I D’Ambrosio G Palmieri VO Moschetta A Palasciano G Portincasa P. Improving nonalcoholic fatty liver disease management by general practitioners: a critical evaluation and impact of an educational training program. JGLD. (2008) 17:389–94. 19104698

[25] Ajzen I . The theory of planned behaviour: reactions and reflections. Psychol Health. (2011) 26:1113–27. doi: 10.1080/08870446.2011.613995 21929476

[26] Montano DE Kasprzyk D. Theory of reasoned actin, theory of planned behaviour, and the integrative model In: Glanz K Rimer BK Viswanath K, editors. Health behaviour and health education 4th edition. San Francisco, CA: John Wiley & Sons Inc. (2008)

[27] Francque SM Marchesini G Kautz A Walmsley M Dorner R Lazarus JV . Non-alcoholic fatty liver disease: a patient guideline. JHEP Rep. (2021) 3:100322. doi: 10.1016/j.jhepr.2021.100322, PMID: 34693236 PMC8514420

