# Supplementary material for: Development and implementation of a continuing medical education program on non-alcoholic fatty liver disease for primary care practitioners in Europe
Source: Front Med (Lausanne). 2023 Mar 23;10:1034626. doi: 10.3389/fmed.2023.1034626 (PMC10077524; doi:10.3389/fmed.2023.1034626)
Supplement: Supplementary file 1 [file Data_Sheet_1.pdf]

Supplementary Material

Supplemental Table 1. Time-points for outcome measurement

Measure (Items)	Pre-training	Post-training	1-month Post-training
Socio-demographics	X		
Knowledge *(7-items)*	X	X	X
Attitudes *(2-items)*	X	X	
Clinical Norms *(3-items)*	X	X	
Confidence *(8-items)*	X	X	X
Clinical behaviours *(4-items)*	X		X
Satisfaction *(6-items)*		X	
Influence on clinical practice *(1-item)*			X

Supplemental Table 2: Changes in primary care provider's NAFLD/NASH related knowledge pre-and post and at the 1-month following feasibility testing of CME intervention

Question/response options	Pre-training *% (n)*	Post-training *% (n)*	1-Month *% (n)*	p-value
**MQ1. Which of the following conditions are associated with NAFLD?**				
a) Obstructive Sleep Apnea	3.6 (1)	0 (0)	0 (0)	
b) Polycystic ovary syndrome	7.1 (2)	0 (0)	0 (0)	
c) Hypothyroidism	10.7 (3)	0 (0)	7.7 (1)	
d) All three of the above*	78.6 (22)	100 (27)	92.3 (12)	
**Correct response:**	**78.6 (22)**	**100 (27)**	**92.3 (12)**	0.097
**MQ2. A 39-year-old man with obesity has recently been diagnosed with NAFLD, based on ultrasound and elevated liver enzymes. What would be the next step in management for you as a PHC professional?**				
a) Recommend weight loss	50.0 (14)	14.8 (4)	30.8 (4)	
b) Evaluate for other causes of liver disease	3.6 (1)	25.9 (7)	7.7 (1)	
c) Refer to specialist for further evaluation	0 (0)	0 (0)	0 (0)	
d) Recommend Vitamin E	0 (0)	0 (0)	0 (0)	
e) All of the above*	46.4 (13)	59.3 (16)	61.5 (8)	
f) None of the above	0 (0)	0 (0)	0 (0)	
**Correct response:**	**46.4 (13)**	**59.3 (16)**	**61.5 (8)**	0.247
**MQ3. Below are statements regarding NAFLD. Which of these is NOT correct?**				
a) Excessive hepatic fat accumulation with insulin resistance	0 (0)	11.1 (3)	15.4 (2)	
b) Steatosis in >10% of hepatocytes*	54.3 (18)	44.4 (12)	38.5 (5)	
c) The exclusion of secondary causes and alcoholic fatty liver disease	0 (0)	15.4 (4)	30.8 (4)	
d) Lipid peroxidation can lead to inflammation and subsequent activation of Kupffer cells	35.7 (10)	25.9 (7)	7.7 (1)	
e) Don't know	0 (0)	3.7 (1)	7.7 (1)	
**Correct response:**	**54.3 (18)**	**44.4 (12)**	**38.5 (5)**	0.135
**MQ4. Which of the following statements is NOT correct?**				
a) NAFLD is a multisystem disease with extrahepatic manifestations	14.3 (4)	7.4 (2)	7.7 (1)	
b) NAFLD only affects the liver and has no effect on other organs*	78.6 (22)	85.1 (23)	76.9 (10)	0.779
c) Extrahepatic metabolic disorders can exacerbate NAFLD/NASH	0 (0)	7.4 (2)	15.4 (2)	
d) The primary treatment is lifestyle intervention.	7.1 (2)	0 (0)	0 (0)	

e) Don’t know	0 (0)	0 (0)	0 (0)	
**Correct response:**	**78.6 (22)**	**85.1 (23)**	**76.9 (10)**	
**MQ5. Which of the following noninvasive tests rule out fibrosis in NAFLD?**				
a) FIB-4 lower than 1.3*	67.8 (19)	59.3 (16)	69.2 (9)	
b) NAFLD fibrosis score: less than 0.05	25.0 (7)	18.5 (5)	15.4 (2)	
c) FIB-4 score: less than 3.25	0 (0)	7.4 (2)	0 (0)	0.368
d) NAFLD Fibrosis Score: less than 0.672	7.1 (2)	0 (0)	0 (0)	
e) Don’t know	0 (0)	14.8 (4)	15.4 (2)	
f) Other	0 (0)	0 (0)	0 (0)	
**Correct response:**	**67.8 (19)**	**59.3 (16)**	**69.2 (9)**	
**MQ6. Which of the following statements is NOT correct? (choose one)**				
a) Lifestyle changes and effective management of metabolic risk factors and comorbidities are the cornerstone of treatment	0 (0)	0 (0)	7.7 (1)	
b) Key to effective treatment is weight loss through lifestyle changes, pharmacotherapy and/or surgical intervention	10.7 (3)	14.8 (4)	0 (0)	
c) Statins are not safe for use in this population and may cause more harm than protection*	85.7 (24)	85.2 (23)	92.3 (12)	0.472
d) A collaborative approach between the specialist, the PCP and the patient is key, including a treatment plan	3.6 (1)	0 (0)	0 (0)	
e) Don’t know	0 (0)	0 (0)	0 (0)	
**f)** Other	0 (0)	0 (0)	0 (0)	
**Correct response:**	**85.7 (24)**	**85.2 (23)**	**92.3 (12)**	
**MQ7. Which of the following lifestyle recommendations for patients with NAFLD is NOT correct? (choose one)**				
a) Aerobic exercise 3 or more days per week	11.1 (2)	3.9 (1)	0 (0)	
b) Reduction in the addition of sugar to the diet	7.1 (3)	7.7 (2)	7.7 (1)	
c) Foods containing saturated fat and cholesterol have no significant effect on NAFLD*	67.9 (19)	69.2 (18)	69.2 (9)	0.472
d) Endurance exercise for 2 or more days/week	14.3 (4)	15.4 (4)	15.4 (2)	
e) Don’t know	0 (0)	3.9 (1)	7.7 (1)	
f) Other	0 (0)	0 (0)	0 (0)	
**Correct response:**	**67.9 (19)**	**69.2 (18)**	**69.2 (9)**	

*indicates correct response

Pre-training data available for n=28 primary care providers, Post-training data available for n=27 primary care providers, I-month follow-up data available for n=13 primary care providers

Overall p-value calculated based on Cochrane’s Q test

Supplemental Table 3: Changes in primary care provider's attitudes and norms related to NAFLD-NASH pre-and post and at the 1-month following feasibility testing of CME intervention

**Measure**	**Pre-training** *% (n)*	**Post-training** *% (n)*	**p-value**
Do you perceive NAFLD as an important public health problem in general?			
Strongly agree	64.3 (18)	57.7 (15)	.655
Agree	28.6 (8)	42.3 (11)	
Neutral	3.6 (1)	0 (0)	
Disagree	3.6 (1)	0 (0)	
Strongly disagree	0 (0)	0 (0)	
Do you perceive NAFLD as an important issue in your own practice?			
Strongly agree	60.7 (17)	57.7 (15)	.705
Agree	32.1 (9)	34.6 (9)	
Neutral	7.1 (2)	7.8 (2)	
Disagree	0 (0)	0 (0)	
Strongly disagree	0 (0)	0 (0)	
**Primary care providers have a central role to play in screening for NAFLD**			
Strongly agree	64.3 (18)	65.3 (17)	1
Agree	28.6 (8)	30.7 (8)	
Neutral	7.1 (2)	3.9 (1)	
Disagree	0 (0)	0 (0)	
Strongly disagree	0 (0)	0 (0)	
**Primary care providers must be up to date on the latest guidelines for NAFLD**			
Strongly agree	57.1 (16)	69.2 (18)	.527
Agree	32.1 (9)	31.0 (8)	
Neutral	10.7 (3)	0 (0)	
Disagree	0 (0)	0 (0)	
Strongly disagree	0 (0)	0 (0)	
**Diagnosis and treatment of NASH/NAFLD is a complex clinical area and should be best managed by specialists vs. general practitioner?**			
Strongly agree	7.1 (2)	3.9 (1)	.819
Agree	3.6 (1)	15.4 (4)	
Neutral	35.7(10)	15.4 (4)	

Disagree	28.6 (8)	38.5 (10)
Strongly disagree	25.0 (7)	26.9 (7)

Pre-training data available for n=28 primary care providers
Post-training data available for n=26 primary care providers
p-values calculated based on data available for PCPs with data at both timepoints
